# Investigation of the gene mutations in two Chinese families with X-linked infantile nystagmus

**Published:** 2011-02-11

**Authors:** Ningdong Li, Xiaojuan Wang, Yuchuan Wang, Liming Wang, Ming Ying, Ruifang Han, Yuyan Liu, Kanxing Zhao

**Affiliations:** 1 Tianjin Medical University, Tianjin, the People’s Republic of China; 2 Tianjin Eye Hospital, Tianjin Eye Institute, Tianjin, the People’s Republic of China; 3 Xuzhou Eye Institute, Xuzhou, Jiangsu Province, the People’s Republic of China

## Abstract

**Purpose:**

To identify the gene mutations causing X-linked infantile nystagmus in two Chinese families (NYS003 and NYS008), of which the NYS003 family was assigned to the FERM domain–containing 7 (*FRMD7*) gene linked region in our previous study, and no mutations were found by direct sequencing.

**Methods:**

Two microsatellites, DXS1047 and DXS1001, were amplified using a PCR reaction for the linkage study in the NYS008 family. *FRMD7* was sequenced and mutations were analyzed. Multiplex ligation-dependent probe amplification (MLPA) was used to detect *FRMD7* mutations in the NYS003 family.

**Results:**

The NYS008 family yielded a maximum logarithm of odds (LOD) score of 1.91 at θ=0 with DXS1001. *FRMD7* sequencing showed a nucleotide change of c. 623A>G in exon7 of the patients’ *FRMD7* gene, which was predicted to result in an H208R amino acid change. This novel mutation was absent in 100 normal Han Chinese controls. No *FRMD7* gene mutations were detected by MLPA in the NYS003 family.

**Conclusions:**

We identified a novel mutation, c. 623A>G (p. H208R), in a Han Chinese family with infantile nystagmus. This mutation expands the mutation spectrum of *FRMD7* and contributes to the research on the molecular pathogenesis of *FRMD7*.

## Introduction

Infantile nystagmus (IN), also referred to as congenital nystagmus (CN), congenital “motor” nystagmus (CMN), idiopathic congenital nystagmus (ICN), or congenital idiopathic nystagmus (CIN), is a relatively common ocular motor disorder characterized by rapid to-and-fro oscillations of the eyes. It usually presents at birth or develops within the first few months of life, and it is different from “sensory defect nystagmus” caused by inherited ocular diseases, including: albinism, achromatopsia, Leber congenital amaurosis, congenital cataract, and anterior segment dysgenesis [1,2]. The etiology of infantile nystagmus is unclear.

IN may be inherited as an autosomal dominant, autosomal recessive, or X-linked trait, while the X-linked inheritance is believed to be the most common mode [3]. Two IN loci have been mapped on chromosome Xp11.4-p11.3 [4] and Xq26-q27 [5], respectively, of which the FERM domain–containing 7 gene (*FRMD7*) was identified to be on the chromosome Xq26-q27 region [6], but no responsible genes have been cloned yet from the Xp11.4-p11.3 region.

**Figure 1:**
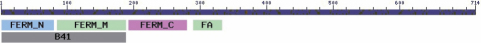
Graphical structure of the FRMD7 domains.

*FRMD7* is a newly identified member of the FERM family (F for 4.1 protein, E for ezrin, R for radixin and M for moesin), and consists of 12 exons spanning approximately 51 kb on chromosome Xq26-q27. *FRMD7* encodes 714 residues and is expressed in the ventricular layer of the forebrain, midbrain, cerebellar primordium, spinal cord, and the developing neural retina in embryos 56 day post-ovulation. It is restrictively expressed in the mid- and hindbrain (where the center of the eye’s movement is located) in earlier embryos 37 day post-ovulation [6]. The FRMD7 protein has B41, FERM-N, FERM-M, FERM-C, and FA structural domains with the conserved domains concentrated at the B41 and FERM-C domains (Figure 1). In fact, the FERM domain is the same as the B41 domain since it was originally identified in band 4.1 (also known as protein 4.1) [7]. The B41 domain is located between residues 1–192, and the FERM-C domain is located between residues 186–279. The FRMD7 protein has close homology to two other FERM domain containing proteins: RhoGEF and pleckstrin domain protein 1 (FARP1; NM_005766) and FARP2 (NM_014808). FARP1 is known to promote the dendritic growth of spinal motor neuron subtypes, while FARP2 has been shown to modulate the length and degree of neurite branching in developing cortical neurons.

We previously reported five mutations in *FRMD7* in seven Chinese families with infantile idiopathic nystagmus [8]. However, no mutations were identified by directly sequencing *FRMD7* in the NYS003 family (one of the seven reported families assigned to the *FRMD7* gene linked region). In this study, we tried to detect *FRMD7* mutations in the NYS003 family by using multiplex ligation-dependent probe amplification (MLPA). We also attempted to identify gene mutations in the other Han Chinese family (NYS008) with infantile nystagmus.

## Methods

### Patient ascertainment

**Figure 2:**
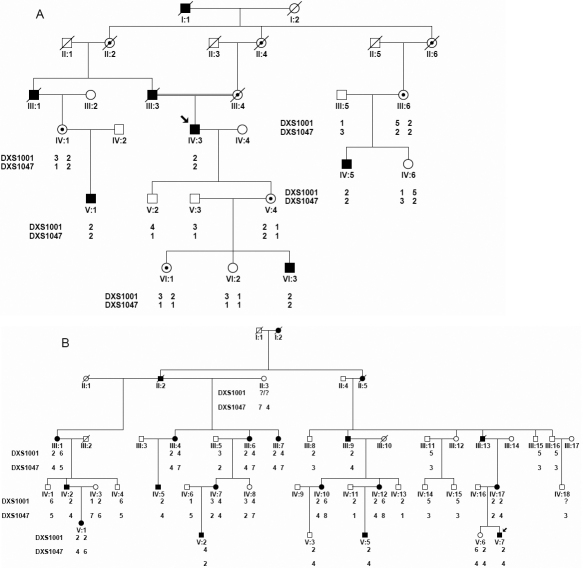
Two Chinese families with X-linked infantile nystagmus. The squares represent the males, the circles represent females, the shaded symbols signify the affected individuals, the dotted circles represent the female carriers, a diagonal line through a symbol indicates a deceased family member, and the arrow indicates the proband.

Two IN families (NYS003 and NYS008) were recruited for this study (Figure 2). All participants underwent an ophthalmologic examination, including visual acuities, slit examination of the lens, examination of the vitreous, fundus, electroretinograms (ERGs), and visual evoked potentials (VEP) as well. Eye movements were recorded with the Eye Tracker system (Eyelink 2000; SR Research, Kanata, Ontario, Canada). After informed consent was obtained, blood samples were collected from the affected and unaffected members in the two IN families. DNA was extracted from blood lymphocytes according to the standard protocol (Roche Biochemical, Inc., Shanghai, China). Briefly, the white blood cells were separated from whole blood via a preferential red blood cell lysis and then lysed by a strong anionic detergent. After the proteins were removed by dehydration and precipitation, the purified DNA is subsequently recovered via ethanol precipitation. This study obtained IRB approval from the Tianjin Eye Hospital, Tianjin, China, and was conducted according to the declaration of Helsinki’s tenets.

### Genotyping and linkage analysis

A linkage analysis was performed with two fluorescently labeled microsatellite markers, DXS1001 and DXS1047, after PCR amplification in a 10 μl reaction mixture containing 50 ng of genomic DNA, 1× PCR buffer, 2.0 mM MgCl_2_, 0.2 mmol/l of each dNTP, 5 pmol/l of each forward and reverse primer, and 0.2 U of Ampli Taq Gold DNA polymerase (Applied Biosystems, Foster city, CA). The PCR products from each DNA sample were pooled and mixed with a loading cocktail containing HD-400 size standards (PE Applied Biosystems, Foster City, CA) and loading dye. The resulting PCR products were separated on an ABI3130 sequencer, and analyzed with GENEMAPPER 3.7 (PE Applied Biosystems).

Two-point logarithm of odds (LOD) scores were calculated with easyLinkage plus v4.0 beta software, assuming an X-linked dominant trait with an affected allele frequency of 0.001. The marker order and distance between DXS1001 and DXS1047 were obtained from the National Center for Biotechnology Information (NCBI).

### Mutation analysis

Mutation screening of *FRMD7* in family NYS008 was conducted using a direct DNA sequence analysis. Individual exons of *FRMD7* were amplified by PCR as previously described [8]. Briefly, whole coding regions and exon-intron boundaries of *FRMD7* were PCR-amplified in 50 μl of standard PCR buffer containing 1.5 mmol/l MgCl_2_, 0.2 mmol/l of each dNTP, 0.5 μmol/l of each primer (Table 1), 1 U of Taq polymerase , and 50 ng of DNA. The amplification program was an initial 2 min denaturation at 98 °C, followed by 30 cycles of 30 s at 94 °C, 30 s at 55 °C, 1 min at 72 °C, and a final 7 min extension step at 72 °C. The PCR products were extracted using the QIAquick Gel Extraction Kit (Qiagen, Valencia, CA). DNA sequencing analysis was performed with the BigDye Terminator Cycle Sequencing V3.1 Kit on an ABI PRISM 3130 Genetic Analyzer (PE Applied Biosystems). Sequencing results were assembled and analyzed with DNASTAR software’s Seqman program (DNASTAR Inc., Madison, WI). A multiple sequence alignment was performed using the Clustal W algorithm in the software package. The modeled structures were built with SWISS-MODEL.

**Table 1:** Primers used to amplify the individual exons of *FRMD7*.

**Exon**	**Forward primer**	**Reverse primer**	**Tm (°C)**	**Product length (bp)**
1	AGGAGACTGCCCAGATGCTA	GGGCTGTTCACAAATGACAA	60	348
2	GAAACAGGGCTTGCAGAGAG	GCAATGCTAGACACAAAGAACC	60	246
3	TGCTCAATGCCATGCTCTAC	AAAGCCCTTTTCTCCCCTTA	58	199
4	TGGGTGTGTGTGTGTGTGTG	GCCCCCAATAAATGGAGAAT	58	237
5	TGGATCTGGGAGAAGGAAAA	GCTCCTGTGCTTGGTTCTCT	60	238
6	GAGGACAAGGGTATGCTGGA	TCAGGTTTAAGGGCTTGCTC	58	281
7	GAGCTCTCAGGGTGGAAATG	ACACCCAAGTTTGAGCCAAG	58	293
8	TGCACTGTCTTACAAGCCAACT	CGATTTGCAGAAACAACCAA	56	231
9	TTGGGATTTGAAGGTCTTTGA	TCCTCCTAAGCCTCCTGTGTT	60	300
10-11	AGCCTATTGGTTTATGGCTAGAAC	GCAGAATCAATTCATGGAAGC	56	393
12a	GGCCTTTTCCTTCTTTCACC	CTGGGGAGGCATAATACCAA	58	472
12b	AGCTCCTTCCAAACAAGCTG	TGACTGAGAGCAGGACAAGG	60	524
12c	GCGGTAGGAGCAACATCAAT	CCAAGAAAATGGTTTCTACAACTTC	58	560

### Multiplex ligation-dependent probe amplification (MLPA)

MLPA was conducted by using the supplied protocol from the SALSA P269 FRMD7-NYS1 probe kit of MRC-Holland (Amsterdam, Holland), which contains 28 MLPA probes with amplification products between 166 and 391 nucleotides. One-hundred ng of DNA (5 μl) was denatured at 98 °C for 5 min, and then was mixed with the probe set and MLPA buffer after cooling at 25 °C. The mixture was re-heated to 95 °C, and then was incubated for 16 h at 60 °C. Following probe hybridization, the DNA ligase and ligation buffer were added, and the ligation was proceeded for 15 min at 54 °C. One microliter of ligation products were amplified by PCR according to the manufacturer’s protocol. The PCR products were then mixed with a loading cocktail containing HD-400 size standards (PE Applied Biosystems) and de-ionized formamide. The resulting PCR products were separated on an ABI3130 sequencer, and analyzed with GENEMAPPER 3.7 (PE Applied Biosystems). Specific peaks corresponding to each exon were identified according to their migration (relative to the size standards), and were exported to a Microsoft Excel (Microsoft Corporation, Redmond, WA) spreadsheet. Peak heights of each fragment were compared to a control sample to obtain a dosage quotient (DQ) representing the gene dosage of each amplicon. For normal sequences, a dosage quotient of 1.0 is expected; if a deletion or duplication is present, the dosage quotient should be 0.7 and 1.3, respectively.

## Results

**Figure 3:**
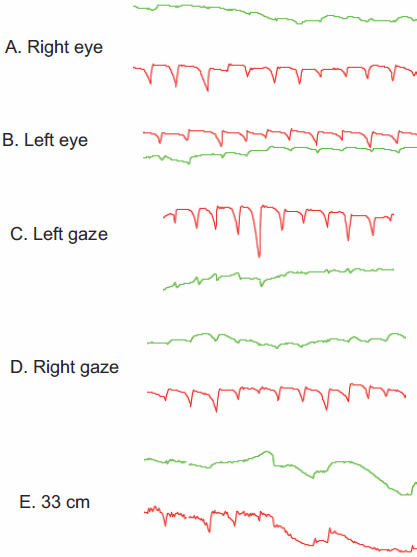
Eye movement recording of the proband in the NYS008 family. Eye movement recordings of the primary gaze of the right and left eyes of the proband showed horizontal nystagmus (**A** and **B**). The ocular oscillation could be dampened at neither the left (**C**) nor the right (**D**) gaze of 10°, but could be achieved at a viewing distance of 33 cm (**E**). The vertical component of the eye movement is denoted in green, and the horizontal movement is shown in red.

The NYS008 family from Hebei province, China, included 4 affected males and 4 female carriers. Individual IV3 was a proband in this family. His parents were consanguineously married (Figure 2A). He developed nystagmus at the age of 3 to 4 months, and had a horizontal jerk ocular oscillation at a distance, with the best-corrected visual acuity of 0.5 in both eyes at the age of 32. His eye movement recordings in a primary gaze of the right and left eyes showed horizontal nystagmus with slow rightward drift and fast beats to the left (Figure 3). The ocular oscillation could be dampened at neither a left nor a right gaze of 10°, but could be achieved at a viewing distance of 33 cm (Figure 3). Other male patients in this family had various reduced visual acuity, ranging from 0.2 to 1.0 with a horizontal jerk ocular oscillation. None of the patients in this family had a compensatory face turn.

**Figure 4:**
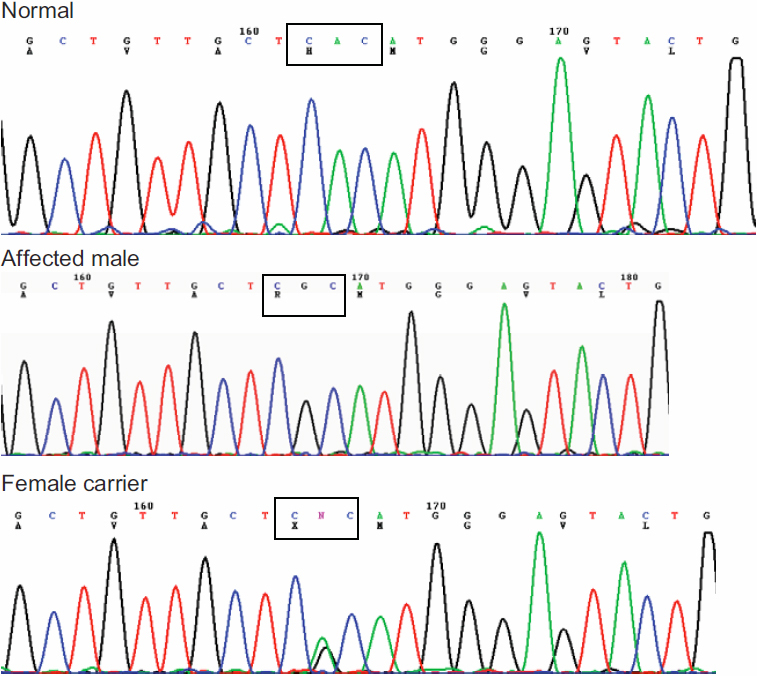
Identification of the 623A>G (p. H208R) in *FRMD7*. Sequencing chromatograms from a normal individual (top), an affected male (middle), and a female carrier (bottom), showing an A→G change in exon 7.

**Figure 5:**
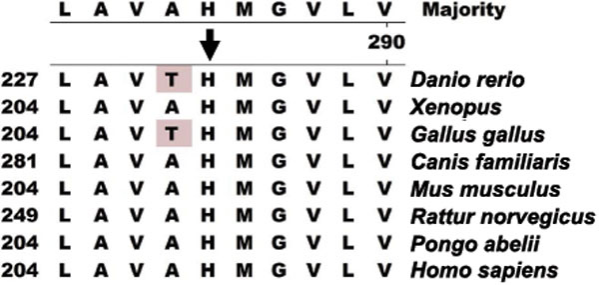
Alignment of FRMD7 amino acids. The alignment of amino acids around p.H208 (denoted by the black arrow) of FRMD7 revealed evolutionary conservation of the Histidine among *Homo sapiens*, *Pongo abelii*, *Rattus norvegicus*, *Mus musculus*, *Canis familiaris*, *Gallus gallus*, *Xenopus lavis*, and *Danio rerio*.

**Figure 6:**
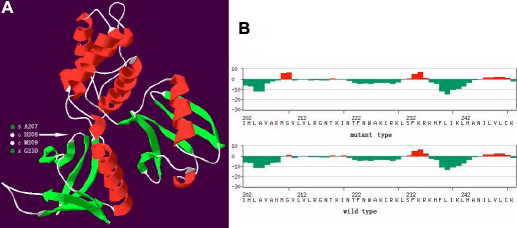
The 3D model and the anolea mean force potential plot for the target protein of human *FRMD7*. **A**: The model rendered in 3-D for the target human FRMD7 protein. α-helices, coils, and β-sheets are depicted in red, gray, and green, respectively. Only two amino acids, H208 and M209, are located inside the region between the two β-sheets (denoted by an arrow). **B**: The anolea mean force potential plot for the target protein of the human FRMD7. Above is the mutant type of the FRMD7 protein; below is the wild type of the FRMD7 protein. The y-axis of the plot represents the energy for each amino acid of the protein chain. Negative energy values (in green) represent a favorable energy environment, whereas positive values (in red) indicate an unfavorable energy environment for a given amino acid. A favorable energy environment tends to be damaged because of an H to R change at codon208.

A linkage study showed that the maximum LOD score of 1.91 was yielded at the polymorphic marker DXS1001 (θ=0), and a positive LOD score of 1.31 was achieved at DXS1047. Sequencing of *FRMD7* illustrated a single nucleotide change of c.623A>G in exon 7, which was predicted to result in a wild type amino acid of Histidine (H) substituted by a mutant type amino acid of Arginine (R) at codon 208 (Figure 4). This nucleotide change was absent in 100 normal controls after sequencing of *FRMD7*. The multiple sequence alignment of the FRMD7 protein indicated that H208R was conserved among *Homo sapiens*, *Pongo abelii*, *Rattus norvegicus*, *Mus musculus*, *Canis familiaris*, *Gallus gallus*, *Xenopus lavis*, and *Danio rerio* (Figure 5). A Position-Specific Independent Counts (PISC) score of 2.53 was yielded after using the POLYPHEN program to predict the functional and structural changes of the amino acid substitution. The modeled protein structure assessed by the ANOLEA program [9] would thus be unstable if the Histidine at codon 208 was substituted by the Arginine (Figure 6).

The NYS003 family reported in our previous study (the C family from the Liaoning province, China [8]), consisted of 6 male patients and 9 female carriers (Figure 2B). The results analyzed by MLPA using 28 probes on the patients are listed in Table 2, and the average dosage quotient (DQ) at each probe ranged from 0.8 to 1.1, which suggested that mutations were not detected by the MLPA method.

**Table 2:** *FRMD7* gene dosage analysis by MLPA in the NYS003 family.

**Probe**	**Average DQ**	**SD**	**dosage**
*PDCD8*	0.9	0.09	Normal
*FRMD7* exon1	1.07	0.07	Normal
*FRMD7* exon1	0.93	0.08	Normal
*FRMD7* exon2	0.81	0.15	Normal
*FRMD7* exon2	0.88	0.09	Normal
*FRMD7* exon4	0.97	0.07	Normal
*FRMD7* exon4	0.83	0.04	Normal
*FRMD7* exon5	0.86	0.07	Normal
*FRMD7* exon6	0.98	0.08	Normal
*FRMD7* exon7	0.98	0.07	Normal
*FRMD7* exon8	0.94	0.07	Normal
*FRMD7* exon9	0.91	0.08	Normal
*FRMD7* exon9	0.98	0.05	Normal
*FRMD7* exon11	1.02	0.03	Normal
*FRMD7* exon12	1.01	0.05	Normal
*FRMD7* exon12	1.03	0.06	Normal
*GPC4*	0.99	0.08	Normal
*GPC3*	1.01	0.08	Normal
Control	0.96	0.11	Normal
Control	1.03	0.08	Normal
Control	1.02	0.04	Normal
Control	1.03	0.07	Normal
Control	0.94	0.08	Normal
Control	0.93	0.06	Normal
Control	1.04	0.08	Normal
Control	1.02	0.07	Normal
Control	0.95	0.07	Normal
Control	1.09	0.06	Normal

For normal sequences, a dosage quotient (DQ) of 1.0 is expected; if a deletion or duplication is present, the dosage quotient should be 0.7 and 1.3, respectively.

## Discussion

To date, 40 mutations in *FRMD7* have been reported worldwide in families with X-linked congenital nystagmus from various ethnic backgrounds [6,8,10-16]. These mutations include missense, nonsense, and splicing mutations, as well as insertions and deletions according to the Human Gene Mutations Database. However, the underlying molecular mechanisms of these mutations in CIN are still not fully understood. *FRMD7* may play an important role in neuronal development, particularly in brain regions that are associated with ocular motor control [17-20]. It may also be involved in signal transduction between the plasma membrane and cytoskeleton because it contains a FERM domain at the NH_2_-terminus [21].

In this study, we found a novel nucleotide change of c.623A>G in exon7 of the *FRMD7* gene in the NYS008 family, but we were unable to detect *FRMD7* mutations in the NYS003 family. The heterozygote change of c.623A>G in *FRMD7* in the NYS008 family should be regarded as a novel gene mutation rather than a polymorphism nucleotide change due to the following reasons: 1) It is absent in 100 normal controls. 2) We predict that the c.623A>G change would result in a Histidine to Arginine change at codon 208 where it is located within highly conserved regions that are invariant in *Homo sapiens*, *Pongo abelii*, *Rattus norvegicus*, *Mus musculus*, *Canis familiaris*, *Gallus gallus*, *Xenopus lavis*, and *Danio rerio*. This suggests that the Histidine at codon 208 is critical to the protein’s normal functioning. 3) A PISC score of 2.53 predicted by POLYPHEN suggests that the substitution of Histidine to Arginine at codon 208 would most likely damage the protein structure and/or the protein function.

Further analysis of the FRMD7 protein structure in the model shows that the Histidine at codon 208 is one of two amino acids located inside the region between the two β-sheets, and substituting it with Arginine is likely to destabilize the protein by introducing a larger amino acid within restricted areas of the protein. This has been demonstrated by the ANOLEA program, which found that a favorable energy environment tends to be damaged by amino acid substitutions in that region (Figure 6).

It has been reported that nearly all missense mutations, except for G296R, Y301C, and S340L, occurred in the B41 and the FERM-C domains of *FRMD7* [6,12]. H208R occurred in the FERM-C domain which seems to be a mutation-rich region [22], and was close to two other known mutations (Q201X and N221D [6]) in the same domain.

The NYS003 family’s responsible gene was previously localized to the region linked with the microsatellite marker DXS1047 (Z_max_=2.42, θ_max_=0.1). No mutations were detected in *FRMD7* by direct sequencing, although all coding regions and exon-intron boundaries were sequenced [8]. The method of direct sequencing based on a single exon's PCR amplification from genomic DNA has several shortcomings in detecting most exon deletions and duplications [23]. MLPA is believed to be a robust assay that offers several advantages for gene mutation detection, particularly for detecting deletions/duplications of one or more exons in the gene [23,24]. It is a variation of the PCR reaction that permits multiple targets to be amplified with only a single primer pair [23]. Each MLPA probe consists of two halves of the oligonucleotides which recognize adjacent target sites on the DNA and can be ligated into a complete probe when both of them are hybridized to their respective targets. Only the ligated oligonucleotides can be amplified, not the unbound probe oligonucleotides. Each complete probe has a unique length so that its resulting amplicons can be separated and identified by electrophoresis. By comparing the peak pattern obtained on a given sample with that obtained on various reference samples, the relative quantity of each amplicon can be determined. Because the amount of ligated probes is dependent on the number of specific primer binding sites, this method is suitable for the detection of chromosomal deletions or duplications.

To exclude the possibility of *FRMD7* gene rearrangements in the NYS003 family, the MLPA method was used in this study. However, we have not yet identified *FRMD7* mutations in this family. The fact that we detected no mutations from either direct sequencing or MLPA, illustrated that our experiments’ mutation screening techniques might be limited in their ability to detect unknown regions (e.g., the promoter or other regulatory regions including those within introns) in *FRMD7*. Further study on the patients’ RNA might find mutations in the unknown regions in *FRMD7*.

Recently, investigations of the molecular pathogenesis to some ocular movement diseases develop very quickly, especially to a group of the congenital cranial dysinnervation disorders (CCDDs), including congenital fibrosis of the extraocular muscles (CFEOM), Duane’s retraction syndrome (DRS), and horizontal gaze palsy with progressive scoliosis (HGPPS) and MÖbius syndrome [25]. Some responsible genes for CCDDs (kinesin family member 21A [*KIF21A*] for CFEOM1 [26], paired-like homeobox 2a [*ARIX*] for CFEOM2 [27], roundabout, axon guidance receptor, homolog 3 [*Robo3*] for HGPPS [28], and chimerin 1 [*CHN1*] for DRS [29]) have been identified and are believed to be involved either in the development of brainstem motor nuclei or in axon guidance. Some advanced neuroimaging techniques, such as diffusion tensor imaging (DTI) and diffusion spectrum imaging (DSI) [30], have also been used to accurately recognize new patterns of aberrant axon connectivity and phenotypically confirm the findings in genetic studies. The combination of advanced neuroimaging techniques and genetic studies is likely to advance our understanding of the molecular mechanisms of congential nystagmus.

In summary, we investigated the gene mutations in two Chinese families with infantile nystagmus and identified a novel gene mutation, c. 623A>G (p. H208R), in *FRMD7* in one of the families. This mutation will expand the mutation spectrum of *FRMD7* and contribute to the study of the *FRMD7* gene’s molecular pathogenesis.
